# The Royal Hospital for Incurables

**Published:** 1893-12-30

**Authors:** 


### THE ROYAL HOSPITAL FOR INCURABLES.

We have recently received communications which show that our strictures on the management of the Royal Hospital for Incurables at Putney, as set forth in our leader of December 2nd, were not only justifiable, but most urgently called for in the interests of the patients. The information which has reached us from within the walls of the institution confirms our view that there is urgent need for reconstruction in the matter of the *personel*. Our informant points out the ease with which the House Committee are kept unaware of the real conditions under which the patients live at Putney. It does not need actual cruelty to make the lives of such helpless beings a burden to them, but such a result is far from the object for which these beneficent institutions were founded and maintained. All kind-hearted Governors should bestir themselves, and by persistent and continuous protest make the further perpetuation of the present evils impossible. We are glad to see *Truth* has called attention to the fatuous folly of the proceedings at the Annual Court, and has pointed out how strongly the conduct of the business and the wording of the report are to be condemned. If *Truth* will institute an independent investigation, as we have done, and publish the result, the lot of these helpless patients will speedily be made better. The following letter justifies our opinion that with the *personel* rests the principal comfort of the patients in such homes. It proves, too, that the same constitution under different management is capable of securing all that could be desired:—

"An Old Frequenter of St. Leonards" writes: From the articles that have appeared from time to time lately in your paper about the Royal Hospital for Incurables, Putney, I think it is only justice to state that their seaside house at St. Leonards, provided by the Governors for the benefit of their patients at Putney, is an undoubted success. It has been my privilege as a visitor to St. Leonards to have paid several visits there, and to have been courteously shown over the house by the kind and efficient Matron, Mrs. Bartholomew, who seems to have the love and confidence of all the inmates, with whom I have had many quiet chats. They all speak of the Matron's devotion and care of them during their stay. The house comfortably accommodates 10 patients; their stay is six weeks, when they are quickly replaced by 10 more. The rooms are large, airy, and well ventilated. The house is in one of the best positions in St. Leonards Marina. Dr. Edgar Duke, one of the leading men at St. Leonards, is hon. physician, and gives his time and skill most unsparingly, and is much appreciated by the patients who from time to time are under his care.

